# Lateral Intercostal Artery Perforator Flap for Enhanced Aesthetic Outcomes in Breast-Conserving Surgery

**DOI:** 10.7759/cureus.82354

**Published:** 2025-04-16

**Authors:** Jonathan Mokhtar, Shaikha Almarzooqi, Sara Tahlak, Veronica Grassi

**Affiliations:** 1 College of Medicine, Mohammed Bin Rashid University of Medicine and Health Sciences, Dubai, ARE; 2 Department of Otolaryngology – Head and Neck Surgery, Dubai Health, Dubai, ARE; 3 Department of Breast Surgery, Mediclinic City Hospital, Dubai, ARE

**Keywords:** aesthetics, breast cancer, breast-conserving surgery, breast reconstruction, licap, oncoplastic

## Abstract

Oncoplastic breast-conserving surgery (O-BCS) combines oncological safety with superior aesthetic outcomes, enabling women to maintain their breast contour following partial mastectomy and other procedures. The lateral intercostal artery perforator (LICAP) flap, a muscle-sparing technique, is a procedure that utilizes excess soft tissue below the armpit to fill the cavity remaining after a lumpectomy for tumors located in the outer quadrant of the breast.

We present an exemplary case of a 45-year-old female who underwent O-BCS for multifocal invasive ductal carcinoma, followed by reconstruction with the LICAP flap. The patient exhibited exceptional aesthetic outcomes following the LICAP flap procedure, preserving a natural breast contour while achieving clear surgical margins and eliminating residual cancer tissue. The efficacy and versatility of the LICAP flap technique qualify it as an effective approach that addresses the reconstructive aspects of oncoplastic breast surgery, while also achieving a successful oncological response.

## Introduction

Globally, breast cancer remains the most frequently diagnosed malignancy among women, with early detection significantly improving outcomes. Breast-conserving surgery (BCS), along with adjuvant radiotherapy, has become the standard of care, offering oncological outcomes comparable to mastectomy while maintaining breast aesthetics [1,2]. However, achieving symmetry can be challenging with larger resections, prompting the use of oncoplastic techniques such as volume displacement and replacement reconstruction [2].

Oncoplastic breast-conserving surgery (O-BCS) merges cancer surgery with aesthetic goals, offering a combination of oncological safety and superior cosmetic outcomes [3]. Volume replacement techniques, such as the lateral intercostal artery perforator (LICAP) flap, utilize autologous tissue to restore breast contour, particularly for tumors located in the lateral breast quadrants. The LICAP flap is favored over alternatives like the thoracodorsal artery perforator (TDAP) flap or latissimus dorsi (LD) flap due to its muscle-sparing properties and minimal donor site complications [4].

This article was previously published as a preprint on the medRxiv server on March 26, 2025.

## Case presentation

A 45-year-old female presented to the oncoplastic breast clinic with complaints of a growing breast lump on the right breast. On examination, a 15 mm palpable lesion at the nine o’clock position was identified in the right breast. Ultrasound revealed two masses: the index lesion measured 11 x 8 mm at the 10 o’clock position, and the second mass measured 9 x 6 mm at the nine o’clock position. Mammography demonstrated extremely dense breast parenchyma, classified as Breast Imaging Reporting and Data System (BI-RADS) density category D by the American College of Radiology (ACR D), with a suspicious asymmetrical density noted in the posterolateral right craniocaudal (RCC) view. Both masses were confirmed as grade 2 invasive ductal carcinomas (IDCs) of no special type (NST), estrogen receptor (ER)-positive (8/8), progesterone receptor (PR)-positive (7/8), HER2-negative, and with Ki-67 of 10-15%. A third MRI lesion was identified, which was confirmed as stromal fibrosis on biopsy, confirming no further malignant involvement. A PET-CT scan was conducted that discerned no fluorodeoxyglucose (FDG)-avid axillary lymph node involvement or distant metastasis.

The patient was planned for a wide local excision, followed by reconstruction using a LICAP flap. Histopathology revealed a 12 mm invasive carcinoma and an 18 mm high-grade ductal carcinoma in situ (DCIS).

**Figure 1:**
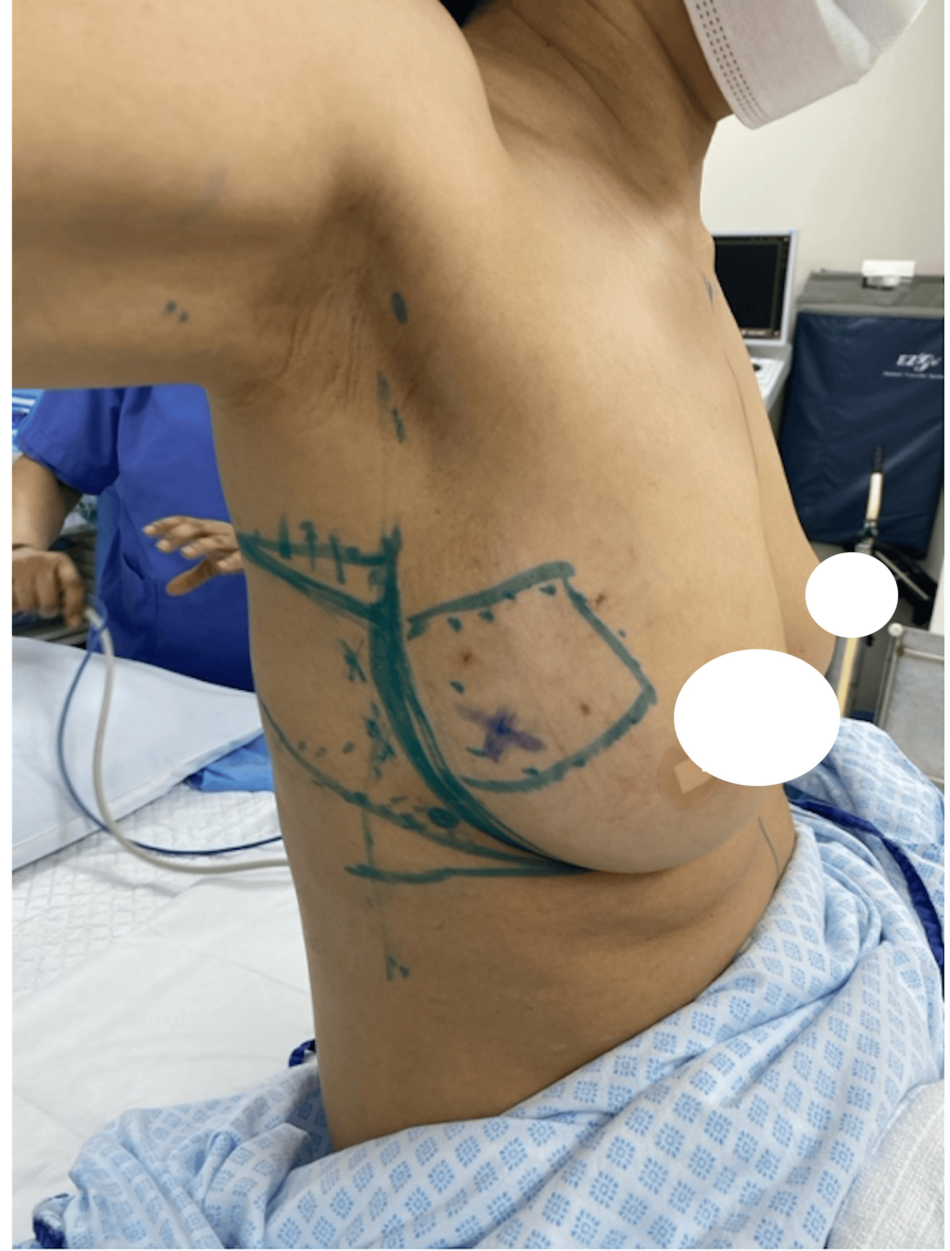
Preoperative image of the breast and planned markings for the LICAP flap. LICAP: lateral intercostal artery perforator.

**Figure 2:**
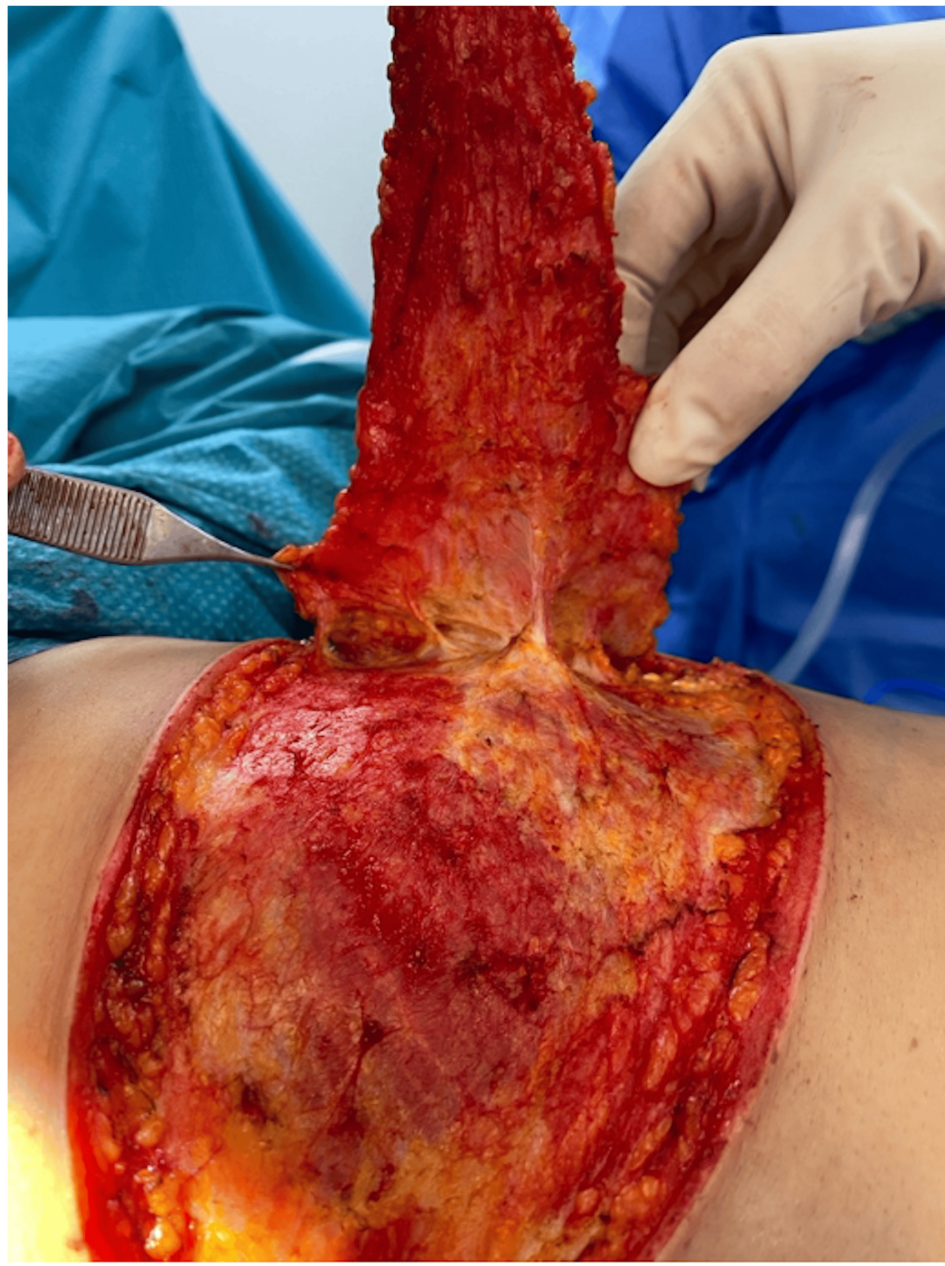
Intraoperative view of the initial dissection. Initial dissection of the lateral intercostal artery perforator flap from the lateral chest wall, illustrating intact perforators.

Preoperatively, the markings of the LICAP flap were carefully planned and drawn along the lateral mammary fold to align with the natural contours of the breast (Figure 1). During the procedure, the incision was made along the lateral mammary fold to align with cosmetic goals. Perforators were identified under direct vision and Doppler guidance, followed by a wide local excision of the tumor with clear margins confirmed by intraoperative Doppler studies to confirm vascular integrity and flap viability. The LICAP flap was harvested from the lateral chest wall, preserving surrounding tissues and ensuring vascularity by keeping the perforators intact (Figure 2). The flap was then rotated into the defect to restore volume and achieve natural breast contour and symmetry.

After a re-excision of the inferior margin to ensure clear margins for the intermediate grade (IG) DCIS, the patient achieved excellent aesthetic outcomes, preserving breast volume and symmetry. Postoperative care included careful monitoring of the surgical site to ensure proper healing and flap viability. A surgical drain was placed to prevent fluid accumulation at the site, and the output was regularly monitored and recorded by the patient to detect any signs of excessive bleeding or seroma formation. Once the output was decreased to an acceptable level, as determined by the surgical team, the drain was removed at the outpatient department. Patient education was one of the most important elements in postoperative care, and the patient was advised to avoid excessive physical activity and strain. She was educated on the red-flag signs, such as bleeding excessively from the surgical site, hematoma formation, surgical site infections, or psychological impact, and instructed to report these immediately.

## Discussion

In modern breast surgery, the focus has evolved from merely achieving oncological control through tumor excision to prioritizing patient satisfaction by improving cosmetic outcomes [4]. Oncoplastic techniques, which integrate tumor removal with reconstruction, aim to enhance the patient’s quality of life while ensuring safe oncological outcomes [4]. In the present case, the patient presented with two multifocal lesions in the upper outer quadrant of the right breast: a 13 mm grade 2 invasive ductal carcinoma and an 8 mm lesion with borderline HER2 status.

Hamdi et al. (2004) first introduced the use of pedicled perforator flaps in breast reconstruction. They investigated various chest wall perforator flaps and highlighted the utility of intercostal artery perforator flaps (ICAP) [5]. The series suggested that the ICAP flap was favorable for patients with defects in the superior and lateral quadrants of the breast, with small to moderate-sized breasts [5]. In alignment with the established framework, the decision to undergo BCS with volume replacement technique using the LICAP flap was influenced by the tumor location, the size of the breast (36C), and the patient’s preference to preserve her breast volume and symmetry.

Oncoplastic techniques are divided into volume displacement and volume replacement procedures [6]. While volume displacement redistributes remaining breast tissue to maintain shape, it is often unsuitable for patients with smaller breasts or tumors that involve a significant portion of breast tissue [6]. The LICAP flap, which involves harvesting skin and fascia from the lateral chest wall, preserves muscle function and offers superior aesthetic outcomes by minimizing donor site morbidity compared to other flap options like the LD flap [6,7].

Several studies highlight the advantages of the LICAP flap in oncoplastic surgery. Orabi et al. (2022) reported that 65.4% of patients who underwent reconstruction with LICAP flaps rated their cosmetic outcomes as excellent, with fewer complications than seen with other flaps like the LD or TDAP flaps [7]. Meybodi et al. (2019) also emphasized that modified LICAP flaps result in better cosmetic outcomes, reduced scarring, and increased patient satisfaction [8]. While TDAP flaps have been associated with complications like venous congestion and fat necrosis, LICAP flaps were simpler to perform, requiring less dissection and carrying a lower risk of partial flap failure [9].

Hashem et al. (2023) compared LICAP and TDAP flaps, finding that LICAP flaps had a lower complication rate (11%) compared to TDAP flaps (17.4%) [9]. LICAP flaps also had a higher proportion of excellent cosmetic outcomes, particularly when used for lateral breast defects [9]. Their use of perforators from the lateral intercostal arteries allows for reliable and consistent results, making the procedure less technically challenging and time-consuming [9]. Mangialardi et al. (2021) further compared LICAP and LD flaps, noting that LICAP flaps preserved chest wall musculature and minimized donor site morbidity, leading to better postoperative outcomes in terms of shoulder function and cosmetic results [10]. This case demonstrates how LICAP flaps provide natural breast contours, meeting the patient’s goals of breast preservation and improving both aesthetic and functional outcomes after BCS.

**Figure 3:**
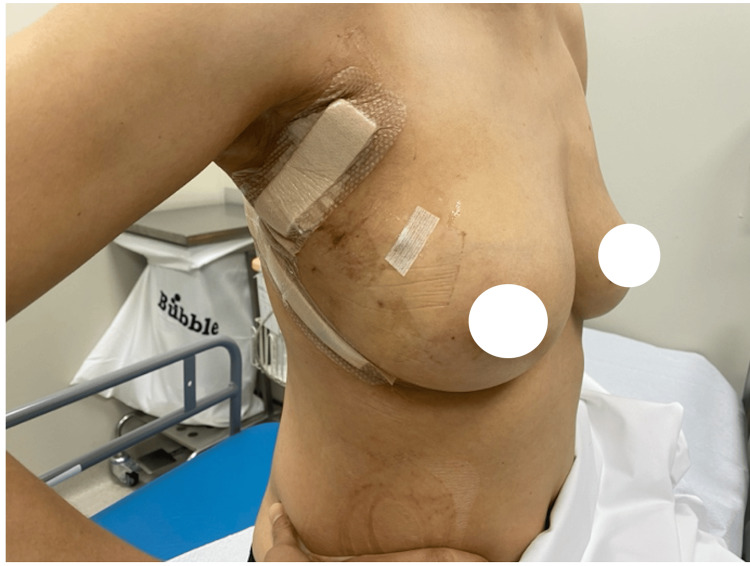
One-month postoperative results. Lateral view at the one-month postoperative visit, demonstrating flap integration and minimal scarring along the lateral mammary fold.

**Figure 4:**
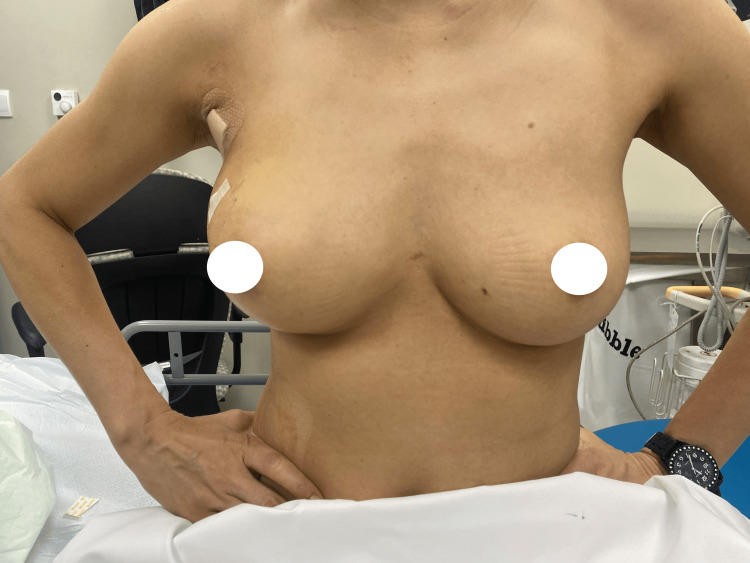
One-month postoperative results. Frontal view at the one-month postoperative visit, demonstrating symmetrical breast shape with maintained volume and contour.

**Figure 5:**
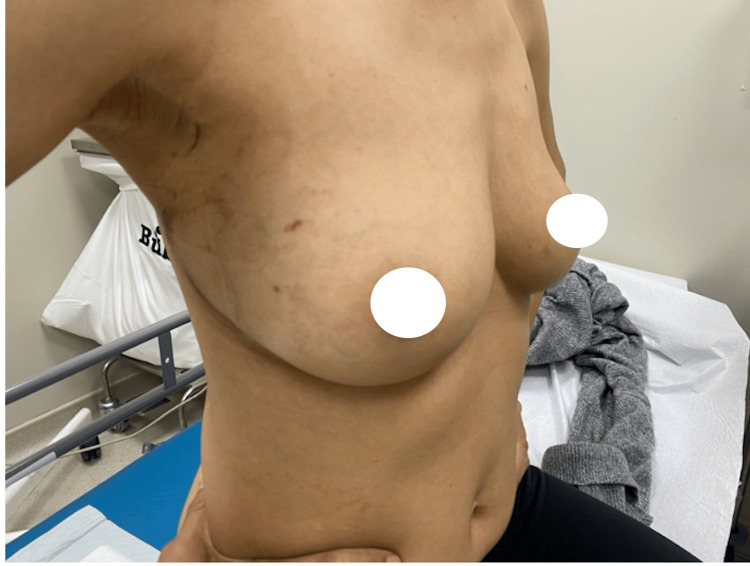
One-year postoperative results. Lateral view at the one-year postoperative visit, demonstrating stable results with inconspicuous scarring and well–preserved breast shape.

**Figure 6:**
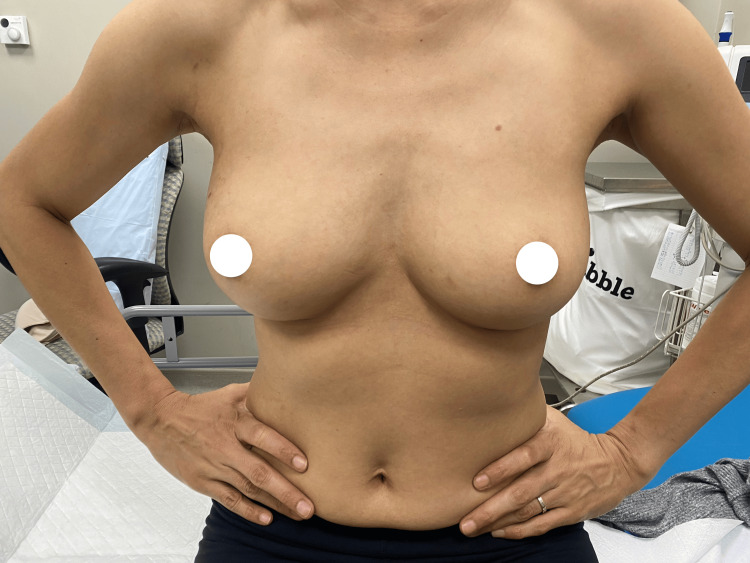
One-year postoperative results. Frontal view at the one-year postoperative visit, demonstrating symmetrical breast shape with well-maintained volume and contour, indicating durable aesthetic results and the absence of postoperative complications.

This case provides a demonstration of the findings discussed above, with the patient achieving remarkable aesthetic outcomes postoperatively. At the one-month and one-year follow-up visits, the patient exhibited excellent restoration of breast contour, volume, and symmetry, with minimal scarring and natural breast shape (Figures 3-6). These postoperative outcomes emphasize the effectiveness of the LICAP flap in providing superior aesthetic results discussed previously.

## Conclusions

The LICAP flap in this case not only allowed for oncological safe tumor removal but also ensured favorable cosmetic outcomes, aligning with the patient’s desire for breast conservation. The reduced donor site morbidity, lower complication rates, and muscle-sparing properties of the LICAP flap make it a valuable tool in oncoplastic breast surgery, emphasizing its role in achieving both oncological and cosmetic goals. The patient expressed high satisfaction with both the cosmetic and functional outcomes, noting minimal discomfort and a natural breast appearance postoperatively. This case exemplifies the current trend in breast cancer surgery to balance oncological safety with patient-centered outcomes, ultimately enhancing the overall quality of life for patients.
